# Effects of GABA-enriched alfalfa silage on rumen microbiota, lactation hormones, immunity, and mammary gland gene expression, alongside lactation performance in dairy goats

**DOI:** 10.1186/s40104-026-01408-9

**Published:** 2026-05-27

**Authors:** Samaila Usman, Jiayao Zhang, Qiang Li, Neha Sheoran, Jing Ma, Dongmei Xu, Demo Joab Usman Kalla, Tunde Adegoke Amole, Xusheng Guo

**Affiliations:** 1 School of Life Sciences, Lanzhou University, Lanzhou, 730000 PR China; 2 Probiotics and Biological Feed Research Center, Lanzhou University, Lanzhou, 730000 PR China; 3 TETFUND Centre of Excellence On Food Security, Dairy Research and Development Centre, Abubakar Tafawa Balewa University, Bauchi, P.M.B. 1048, Bauchi, Nigeria; 4 International Livestock Research Institute (ILRI), IITA Campus P.M.B. 5320, Oyo Road, Ibadan, Nigeria

**Keywords:** Alfalfa silage, Dairy goats, γ-Aminobutyric acid (GABA), Gene expression, Immunity, Lactation hormones, Rumen microbiota

## Abstract

**Background:**

Gamma-aminobutyric acid (GABA) influences metabolic homeostasis, immune function, and lactation performance. Typically, GABA is administered exogenously, but this approach is limited by intake variability and cost. Alternatively, silage inoculated with high GABA-producing *Lentilactobacillus buchneri* YM9 enriches the silage with GABA and ensures more uniform dietary delivery. However, the effects of silage-based GABA enrichment and delivery on ruminant performance, immunity, and health remain unclear. Hence, we conducted a feeding trial with 36 dairy goats assigned to three TMR treatments containing alfalfa silage: 1) CK (uninoculated control), 2) AH35 (inoculated with non–GABA-producing *Lent. buchneri* AH35), and 3) YM9 (inoculated with GABA-producing *Lent. buchneri* YM9). Feed intake and lactation performance, rumen fermentation and microbiota, blood GABA and lactation hormones, blood cytokines and interleukins as well as milk GABA and mammary gland gene expression were evaluated.

**Results:**

YM9-fed goats had lower DMI (1.66 kg/d) compared with CK (1.84 kg/d) and AH35 (1.84 kg/d) but maintained milk yield comparable to CK and higher than AH35 (*P* < 0.05). GABA intake, and milk yield, fat, protein, lactose, total solids, and total nitrogen per kilogram DMI were higher in YM9 (*P* < 0.05). Milk GABA was greater in YM9 (152 μmol/L) than CK (114 μmol/L) and AH35 (111 μmol/L) (*P* < 0.01). Serum GABA (1.44 μmol/L), prolactin (7.65 ng/mL), and oxytocin (4.75 pg/mL) were elevated in YM9 (*P* < 0.05). Immunoglobulins were higher in AH35 overall, but YM9 exceeded CK (*P* < 0.05), with cytokine profiles in the YM9 group reflecting moderate pro-inflammatory activation. YM9 upregulated *GSR* and downregulated *NOX4*, *TNF*, and *IFNG* (*P* < 0.05). Microbiota analysis showed comparable alpha diversity between YM9 and CK with *Prevotella* dominance in the YM9 group, and significant correlations among GABA, microbial taxa, hormones, and mammary gland genes.

**Conclusion:**

Feeding dairy goats with GABA-enriched silage was associated with improved feed and lactation efficiency. It also enhanced lactation hormones, immune responses, and expression of antioxidant-related gene alongside reduced inflammatory related genes. These findings provide alternative approach for dietary GABA delivery to ruminants for enhanced productivity and health, although further studies are required to verify the mechanisms.

**Supplementary Information:**

The online version contains supplementary material available at 10.1186/s40104-026-01408-9.

## Introduction

Gamma-aminobutyric acid (GABA) is a non-protein amino acid classically known as a principal inhibitory neurotransmitter in the central nervous system, but mounting evidence shows that it exerts diverse systemic effects beyond neurotransmission [1, 2]. GABA has been reported to influence thermoregulation, appetite, hormone secretion, stress responses, enhance immunity and upregulates antioxidant defenses by influencing the elevation of antioxidant enzyme activities especially in stressed animals [1, 2]. GABA directly modulate immune cells by altering cytokine secretion and cell proliferation [3, 4]. Together with its receptors, GABA is expressed on various immune cell types, where GABAergic signaling can modulate cytokine production, immune cell proliferation, and inflammatory pathways, including NF-κB [5, 6]. Studies suggest that GABA may support immune homeostasis by promoting regulatory cytokine profiles and antibody-mediated responses, such as increased IgA, IgM, and Th2-associated cytokines, while limiting excessive pro-inflammatory activation [7, 8]. Therefore, GABA contributes to physiological resilience beyond its neural role, with potential implications for systemic immunity and tissue-specific functions such as mammary gland health. Moreover, fermented feeds or silages that generate GABA during microbial fermentation may represent a practical route for delivering GABA to livestock even though its biological implications in ruminant systems is still underexplored.

Evidence from various feeding trials with GABA corroborates its physiological functions. For instance, heat-stressed dairy cows supplemented with rumen-protected GABA had significantly increased antioxidant enzymes (superoxide dismutase and glutathione peroxidase) and immunoglobulins (IgA, IgG), while reducing pro-inflammatory cytokines (IL-2, IL-4, IL-6, TNF-α) and markers of lipid peroxidation (malondialdehyde) [9]. Consistent with improved resilience, GABA-fed cows also showed higher dry matter intake (DMI) and milk protein yield under heat stress [1]. Similar benefits occur in other species, where dietary supplementation of GABA improved performance, inhibited activities of intracellular enzyme in the serum, and protected the morphology of organs and intestine of heat-stressed poultry [10]. Therefore, GABA supplementation may also affect gene expression in tissues such as the mammary gland, as there is evidence indicating its ability to modulate antioxidant and immune-related pathways, which are essential for maintaining udder health and facilitating milk synthesis.

GABA supplementation is delivered exogenously in most studies with ruminants [9, 11, 12]. However, this approach requires additional resources for preparation and delivery of GABA as direct feed additive. To reduce the cost and other extra resources required, we have earlier demonstrated that inoculating corn and alfalfa silages with a high GABA-producing *Lentilactobacillus buchneri* strain substantially enrich the silage with GABA [13]. Therefore, as silage is the major feed for ruminants, especially dairy animals, feeding GABA enriched silage provides an alternative way to deliver GABA to the animals, and this study is the first to deliver GABA to ruminant livestock via silage. Moreover, an in vitro study found GABA to influence volatile fatty acid (VFA) production [14] suggesting that feeding GABA-enriched silage could alter rumen fermentation profiles and potentially modulate microbial community composition to improve feed and nutrient utilization efficiency.

Lactation performance in dairy animals is closely associated with rumen fermentation, systemic immune status, physio-hormonal regulation, and mammary gland health. During the periparturient period, elevated oxidative stress can impair antioxidant defenses and increase susceptibility to mastitis, resulting in reduced milk yield and quality [15–17]. GABA possesses antioxidant and immunomodulatory properties and may therefore influence rumen function, systemic immunity, and lactation outcomes. Building on our recent silage-based microbial enrichment of GABA, this study addresses whether silage-based GABA delivery can beneficially modulate rumen fermentation and microbiota while enhancing antioxidant and immune status to support lactation performance. Accordingly, we hypothesized that feeding GABA-enriched silage would improve rumen fermentation and microbial composition, feed efficiency, milk yield and quality, as well as antioxidant and immune responses in dairy goats.

Therefore, this study aimed at determining the feed intake, yield and quality of milk, rumen microbiota and fermentation profile, blood GABA and lactation related hormones, blood cytokines and interleukins as well as milk GABA concentration and mammary gland gene expression of lactating dairy goats fed *Lent. buchneri* YM9 inoculated GABA-enriched alfalfa silage.

## Materials and methods

### Ethic statement

The animal protocols adopted in the entire study were strictly according to the ethical guidelines accented by the School of Life Science Ethical Committee (No. EAF2024027), Lanzhou University.

### Experimental silage

Fresh alfalfa (*Medicago sativa* L. cv. Zhongmu No. 1‌) was harvested mechanically at flowering stage on 13^th^ June 2024. It was wilted to 38% DM and mechanically cleaved into 2–3 cm length. Prior to ensiling in a wrapped bale silo of 55–60 kg, the cleaved forage was thoroughly mixed using a mini loader equipment and randomly divided into three equal portions. Each portion was inoculated with either *Lent. buchneri* YM9 (YM9 treatment), or *Lent. buchneri* AH35 (AH35 treatment) or sprayed with equal amount of water (CK). The inoculant strains were obtained from our laboratory collection, and their isolation, identification, and screening procedures as well as the powder production have been reported previously [13]. Prior to application, inoculant purity and activity were verified using de Man Rogosa Sharpe (MRS) agar. Viable colony count was conducted, and the inoculants were applied at a rate of 1 × 10^5^ colony-forming units (CFU)/g fresh weight (FW). For application, the powdered inoculant was diluted in a 20-L capacity knapsack sprayer and applied to the wilted alfalfa as described by Zhang et al. [17]. The silos were kept for a 60-d fermentation period in a dry place under shed prior to feeding.

### Experimental diets, animal management, and sample collection

Three TMR treatments were formulated using silage from the three inoculation treatments CK, AH35 and YM9. The ingredient composition and nutrient profile of the formulated diets are summarized in Table 1, and they were balanced to meet the requirements for mid lactating dairy goats [18]. The silages' fermentation characteristics are presented in Table S1 (Additional file 1). During the feeding trial, once a silage bale is opened, 100 g sample was immediately collected and preserved at −20 °C. Samples from the concentrate were obtained weekly, and both the concentrate and silage samples were subsequently homogenized based on the respective treatments, with each having five replications.

**Table 1:** Experimental TMR composition, nutrient profile and silage GABA concentration

Item	Inclusion level			SEM	*P*-value
	CK	AH35	YM9		
Ingredients, %					
Alfalfa silage	55	55	55	-	-
corn grain	25	25	25	-	-
Flaxseed	4	4	4	-	-
Wheat bran	13	13	13	-	-
Sodium bicarbonate	1	1	1	-	-
Premix	2	2	2	-	-
Chemical composition, %					
CP	13.48	12.60	12.40	0.27	-
aNDF	31.75	32.25	34.09	0.71	-
ADF	20.60	22.43	24.54	0.44	-
Silage GABA, g/kg DM					
60 d	3.81^c^	3.99^b^	4.24^a^	0.04	< 0.001
90 d	6.06^b^	5.37^c^	6.99^a^	0.15	< 0.001
Composite	5.68^b^	4.87^c^	6.81^a^	0.24	0.001

^a,b^ Means on the same row having different superscripts are significantly different (*P* < 0.05)

A total of 36 multiparous Guanzhong dairy goats (body weight (BW) = 41.07 ± 5.00 kg) at the mid-to-late lactation stage with an average daily milk yield of 0.67 kg/d were used in this study. The dairy goats were housed individually in pens measuring approximately 1.1 m × 1.5 m (1.65 m^2^ per animal), which meets recommended space allowances for adult dairy goats. The pens were demarcated from a well-ventilated, semi-open-sided barn that allowed adequate natural airflow and lighting. The floor was concrete and maintained in a clean and dry condition, with regular removal of manure to ensure hygiene. Animals had free access to clean drinking water throughout the experimental period.

The animals were considered experimental units, and initially offered the control (CK) diet for 10 d to establish baseline BW, DMI and milk yield. Based on these measurements, animals were blocked and then randomly allocated to three dietary treatments, with 12 goats per treatment, using the *blockTools* package in RStudio. The average baseline BW, DMI, and milk yield after allocation were 40.02 kg, 1.76 kg/d, and 0.68 kg/d for CK; 41.77 kg, 1.75 kg/d, and 0.67 kg/d for AH35; and 39.89 kg, 1.70 kg/d, and 0.69 kg/d for YM9, respectively. Post-randomization diagnostics confirmed that treatment groups were balanced with respect to the baseline variables used for blocking.

Feeds were provided to the individual animals ad libitum after milking, with 60% offered in the morning and 40% in the evening. After a 14-d adaptation period, records of daily feed offered, refusals, and milk yield were collected for 21 d. Milk samples were obtained at 7 d intervals, pooled by milking time (morning:afternoon = 6:4), and preserved with bronopol at 4 °C prior to analysis [17].

On the final day of the trial (d 45), animals were weighed before the morning feeding to determine body weight changes during the experimental period. Rumen fluid was collected from each goat using an esophageal tube, with the initial 20–30 mL discarded to minimize saliva contamination. The samples were then filtered through four layers of cheesecloth and stored at −20 °C until analysis. Blood samples were obtained via jugular venipuncture using scalp vein needles into anticoagulant-free vacutainer tubes (Kangjian Medical Products Co., Ltd., Jiangsu, China). The blood was centrifuged at 1,800 × *g* for 15 min at 4 °C, after which the serum was transferred into 2-mL tubes and stored for subsequent analyses.

Mammary gland tissue was obtained by needle biopsy as previously described [17]. Approximately 15 min before the procedure, goats were administered xylazine hydrochloride intravenously and procaine subcutaneously (both from Jilin Huamu Animal Health Products Co., Ltd., Changchun, China) for sedation and local anesthesia. The collected tissue samples were immediately placed into 2-mL cryogenic tubes containing ~ 1.5 mL of RNA stabilization solution and stored for subsequent RNA extraction.

### Laboratory analysis of the samples

#### Fermentation parameters and chemical composition

Silage juice was obtained by homogenizing 20 g of fresh silage with 180 mL of distilled water using a high-speed electric blender for 30 s and filtering the homogenate through four layers of medical gauze. pH was determined immediately. Subsequently, organic acid concentrations were determined by high-performance liquid chromatography (HPLC, 1200, Agilent Technologies, Inc., Santa Clara, CA, USA) as described previously [19]. Silage GABA concentration was also determined from the silage juice as described previously [13]. The TMR samples were oven-dried at 65 °C for 72 h to determine DM content and then ground to pass through a 1-mm screen. Crude protein (CP; calculated as total nitrogen × 6.25) and ash contents were analyzed following AOAC procedures [20]. Neutral detergent fiber (aNDF), determined with heat-stable α-amylase and sodium sulfite, and acid detergent fiber (ADF) were measured using the van Soest method [21] with an Ankom fiber analyzer (A2000I, Ankom Technology, Fairport, NY, USA).

#### Milk quality and rumen fermentation profile

Automatic milk composition analyzer (MilkoScan FT1, Foss, Hillerød, Denmark) was used to determine the fat, protein, lactose, casein, total solid (TS), and solids-not-fat (SNF). Milk GABA concentration was determined according to Usman et al. [13]. Rumen fluid pH was measured immediately after sampling using a portable pH meter (PB-10, Sartorius, Goettingen, Germany). Aliquots of the rumen fluid were subsequently analyzed for volatile fatty acids (VFAs) and ammonia nitrogen (NH_3_-N) following the methods described by Li et al. [22] and Broderick and Kang [23], respectively. Microbial protein (MCP) was determined using total protein quantification assay kit (Nanjing Jiacheng Bioengineering Institute, Nanjing, China; Product No. A045-4-2).

#### Serum immunoglobulins and interleukins

Serum concentrations of immunoglobulins (IgA, IgG, and IgM), interleukins (IL-1β, IL-2, IL-4, IL-6, and IL-10), interferon-γ (IFN-γ), tumor necrosis factor-α (TNF-α), as well as GABA, prolactin, and oxytocin were quantified using commercially available goat-specific enzyme-linked immunosorbent assay (ELISA) kits (Shanghai Yuanju Biotechnology Center, Shanghai, China). Each sample was analyzed in duplicate, and the product catalog numbers of all kits are provided in Additional file 1: Table S2.

### RNA extraction and mRNA expression analysis

Total RNA was isolated using the TRIzol Plus RNA Purification Kit (Thermo Fisher Scientific, USA). RNA concentration and purity were assessed using a NanoDrop ND-2000 spectrophotometer by determining the 260/280 nm absorbance ratio, and integrity was confirmed on 1% agarose gels by visualizing distinct 18S and 28S rRNA bands with an ImageQuant LAS 400 system (GE Healthcare). First-strand cDNA was synthesized using a reverse transcription kit (Takara, Beijing, China).

Quantitative real-time PCR was carried out on a QuantStudio 5 Real-Time PCR System (Applied Biosystems, Thermo Fisher Scientific) to evaluate the expression of 10 target genes, including NADPH oxidase 4 (*NOX4*), tumor necrosis factor (*TNF*), interferon gamma (*IFNG*), nuclear factor erythroid 2-related factor 2 (*NRF2*), superoxide dismutases (*SOD1*, *SOD2*), glutathione peroxidases (*GPX1*, *GPX2*), catalase (*CAT*), and glutathione reductase (*GSR*). *GAPDH* was used as the internal reference. Primers were designed based on NCBI reference sequences and synthesized by Tsingke Biotechnology Co., Ltd. (Additional file 1: Table S3). PCR reactions were prepared in a 20 µL total volume using TB Green^®^ Premix Ex Taq™ II (2 ×) (Tli RNaseH Plus, Cat. RR820A; Takara, Beijing, China), containing 10 µL premix, forward and reverse primers at final concentrations of ~ 0.2–0.4 µmol/L each, ~ 2 µL of cDNA template, and nuclease-free water to volume (following the manufacturer’s recommended setup for Applied Biosystems systems). The cycling program consisted of an initial denaturation at 95 °C for 10 min, followed by 40 cycles of 95 °C for 15 s and 60 °C for 30 s, with a post-amplification melt curve stage to verify specificity. All samples were run in triplicate, and relative gene expression was calculated using the 2^−ΔΔCt^ method [17].

### Extraction of microbial DNA and 16S rRNA analysis

Microbial DNA was extracted from the rumen fluid using a commercial kit (TianGen Biotech Co. Ltd., Beijing, China) and the quantity and quality were evaluated using Nanodrop (Thermo Fisher Scientific, Madison, WI, USA) and 1.2% agarose gel, respectively. After PCR amplification of the 16S rRNA gene region, the amplicon was verified on a 2% (w/v) agarose gel purified using an AxyPrep DNA Gel Extraction Kit (Axygen Biosciences, Union City, CA, USA). The DNA amplicons were quantified using a Quantus™ Fluorometer (Promega, Madison, WI, USA) before DNA sequencing on an Illumina platform (NovaSeq250) made available by BioDeep (Suzhou Panomic Biomedical Technology Co., Ltd., Suzhou, China). QIIME2 software was used for the processing, clustering and taxonomic classification of the sequences. UPARSE (Version 7.1) was employed for the clustering of operational taxonomic units (OTU) with 97% similarity and simultaneously eliminating chimeric DNA sequences.

### Data analysis

The various data analyses were conducted in RStudio of Posit statistical software and figures were generated using ggplot2 package. Data on silage fermentation characteristics and nutrient profile of the TMR were subjected to one-way ANOVA. All other data (except bacterial community) were analyzed by mixed model (with fixed effects being the different inoculation treatments and random effects being the experimental animals) using lme4 package. To analyze daily milk yield and DMI, recording date was added as fixed effect to account for repeated records. Tukey HSD was employed for mean separation. *P*-values were corrected for multiple testing using Benjamini–Hochberg false discovery rate (FDR) in lmerTest package, and significance was declared at 95% probability level.

The statistical analysis of bacterial community (alpha and beta diversity) was conducted using Microeco package [24]. Alpha diversity indices were estimated and visualized while distinct and shared bacterial OTU among the treatments were visualized by a Venn diagram. Subsequently, principal coordinate analysis (PCoA) was used to visualize bacterial OUT-based community differences among treatments. Relative abundance (%) of the bacterial genera were visualized using stacked bar plots, and combination of linear discriminant analysis (LDA) and effect size measurement (LEfSe) was employed to identify bacterial genera with significant (*P* < 0.05) abundance among the treatments at a threshold of 0.001. To control for multiple comparisons across taxa, *P*-values from the statistical tests were adjusted using the Benjamini–Hochberg FDR procedure. Spearman correlation and Mantel’s test analysis were conducted using linkET and vegan packages in Rstudio.

## Results

### Dairy goats’ performance and milk components

The DMI of the dairy goats was highest (*P* < 0.05) in the CK group (1.84 kg/d), followed by AH35 group (1.78 kg/d), then YM9 group which had the lowest (1.66 kg/d) DMI (Table 2). Despite having significantly lower DMI, the daily milk production recorded in the YM9 group was comparable to that of the CK group, whereas the AH35 group had the least daily milk yield (*P* < 0.05). Notably, milk produced per kilogram of DMI was significantly higher in the YM9 goats (*P* < 0.05), indicating superiority in feed conversion efficiency. Correspondingly, the YM9 group had the highest GABA intake (6.26 g/d), followed by CK (5.73 g/d) and lastly the AH35 group (4.78 g/d) (*P* < 0.05). The AH35 and YM9 groups produced significantly higher milk fat, protein, and casein per kilogram of DMI than the CK group (*P* < 0.05). The YM9 group had the highest lactose, TS and SNF per kilogram of DMI (*P* < 0.05), which further supports the improved utilization efficiency associated with YM9-inoculated silage. The goats’ milk of the YM9 group had a significantly higher GABA concentration (152 μmol/L) while CK and AH35 groups had significantly lower GABA concentrations of 114 μmol/L and 111 μmol/L, respectively (*P* < 0.05).

**Table 2:** Dairy goats’ performance and milk components

Item	Treatments			SEM	*P*-values
	CK	AH35	YM9		
DMI, kg/d	1.84^a^	1.78^b^	1.66^c^	0.010	< 0.001
Weight gain, kg	4.31	5.91	5.95	0.83	0.291
GABA intake, g/d	5.73^b^	4.78^c^	6.26^a^	0.117	< 0.001
Milk production					
Milk yield, kg/d	0.64^a^	0.58^b^	0.64^a^	0.016	0.008
Milk yield, g/kg DM	350^b^	331^b^	383^a^	9.210	< 0.001
ECM, kg/d	0.38	0.42	0.45	0.049	0.648
ECM, g/kg DM	209	241	267	28.7	0.367
Fat, g/kg DM	9.78^b^	12.5^a^	13.4^a^	0.334	< 0.001
Protein, g/kg DM	12.5^b^	14.4^a^	15.2^a^	0.365	< 0.001
Lactose, g/kg DM	15.2^b^	14.9^b^	17.1^a^	0.428	0.001
Casein, g/kg DM	10.5^b^	11.9^a^	12.8^a^	0.305	< 0.001
TS, g/kg DM	39.7^c^	43.8^b^	48.0^a^	1.130	< 0.001
SNF, g/kg DM	31.5^b^	33.2^b^	36.9^a^	0.879	< 0.001
GABA, μmol/L	114^b^	111^b^	152^a^	3.99	< 0.001

^a–c^ Means on the same row having different superscript are significantly different (*P* < 0.05)

*SEM* Standard error of the mean. Values represent the mean of 12 samples (*n* = 12)

*DMI* Dry matter intake, *ECM* Energy corrected milk, *TS* Total solid, *SNF* Solid not fat

### Dairy goats’ rumen fermentation profile

The rumen pH was similar among CK, AH35 and YM9 groups (Table 3). Similarly, the TVFA, acetate, propionate, butyrate and isobutyrate of the dairy goats in all the treatments were not statistically different (*P* > 0.05). However, valerate and isovalerate were significantly different among the treatments (*P* < 0.05). The goats in the YM9 and CK groups had higher rumen isovalerate than those in AH35 groups while the CK group had higher rumen valerate concentration than YM9 and AH35 groups. Notably, isobutyrate is at a threshold of statistical significance (*P* = 0.05), hence the difference among treatment groups was considered marginal.

**Table 3:** Dairy goats’ rumen fermentation profile

Item	Treatments			SEM	*P*-values^*^
	CK	AH35	YM9		
pH	7.25	7.2	7.2	0.04	0.623
TVFA, mmol/L	44.21	45.56	38.99	2.55	0.211
Acetate, mmol/L	32.29	33.72	28.31	2.00	0.198
Propionate, mmol/L	6.29	6.27	5.31	0.45	0.263
Butyrate, mmol/L	3.47	3.64	2.97	0.19	0.075
Valerate, mmol/L	0.32^a^	0.29^b^	0.25^b^	0.02	0.040
Isobutyrate, mmol/L	0.78	0.74	0.83	0.03	0.053
Isovalerate, mmol/L	1.04^a^	0.91^b^	1.02^a^	0.03	0.012
NH_3_-N, mg/dL	7.37	7.34	6.96	0.40	0.732
MCP, mg/dL	56.10	51.10	58.40	3.00	0.165

^a,b^ Means on the same row having different superscripts are significantly different (*P* < 0.05)

*SEM* Standard error of the mean. Values represent the mean of 12 samples (*n* = 12)

^*^P-values are FDR adjusted

### Dairy goats' serum contents of GABA, prolactin and oxytocin

**Fig. 1:**
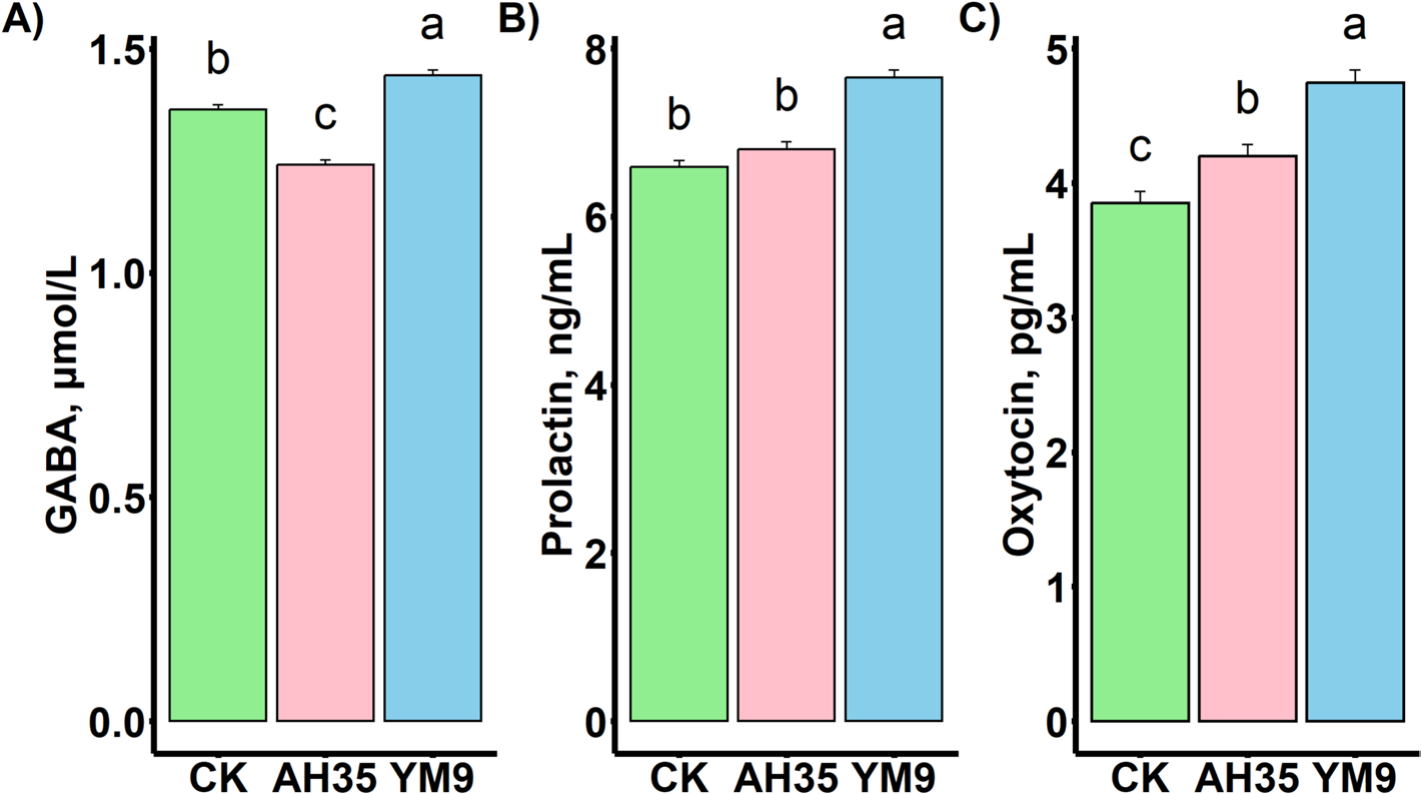
Dairy goats' serum contents of GABA (**A**), prolactin (**B**) and oxytocin (**C**) *n* = 12. Different letters (a, b, c) above bars indicate significant differences (*P* < 0.05)

The YM9 treatment which had the highest silage GABA and daily GABA intake, recorded a significant (*P* < 0.05) concentration (1.44 μmol/L) of GABA in the serum compared with CK (1.36 μmol/L) and AH35 (1.24 μmol/L) treatments (Fig. 1). The serum GABA content of the CK group was significantly higher than that of AH35 group (*P* < 0.05). Similarly, the serum of the goats in the YM9 treatment had significantly (*P* < 0.05) higher prolactin and oxytocin (7.65 ng/mL and 4.75 pg/mL, respectively). The CK and AH35 groups had statistically similar and lower concentration of prolactin (6.59 and 6.83 ng/mL, respectively). The oxytocin concentration in the AH35 group (4.20 pg/mL) was higher than in the CK group (3.85 pg/mL) (*P* < 0.05).

### Serum immunoglobulins and inflammatory cytokines in dairy goats

**Fig. 2:**
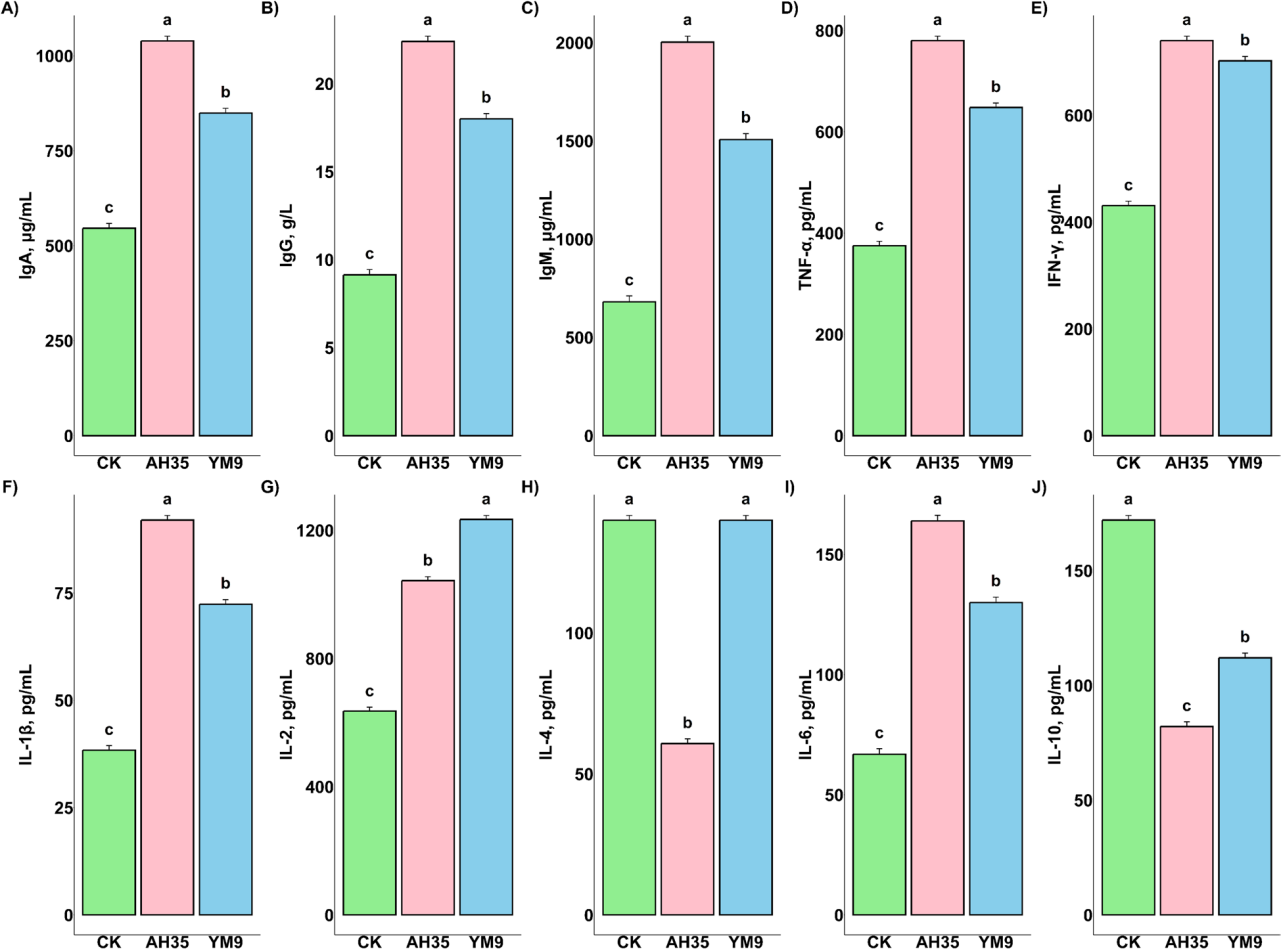
Dairy goats’ serum immunoglobulins and inflammatory cytokines (**A**–**J**) *n* = 12. Different letters (a, b, c) above bars indicate significant differences (*P* < 0.05)

The dairy goats’ serum immunoglobulins as well as pro- and anti-inflammatory cytokines are presented in Fig. 2. The figure revealed that the immunoglobulins IgA, IgG and IgM were significantly higher in AH35 (1,039 μg/mL, 22.4 g/L, and 2,003 μg/mL) and YM9 (894 μg/mL, 18.0 g/L, and 1,507 μg/mL) than CK (546 μg/mL, 9.14 g/L, and 681 μg/mL) (*P* < 0.05). Similarly, goats in the AH35 and YM9 group had significantly higher pro-inflammatory cytokines TNF-α (780 and 648 pg/mL), IFN-γ (740 and 702 pg/mL), IL-1β (92.0 and 72.4 pg/mL) and IL-6 (164 and 130 pg/mL) while CK had the least concentration (375, 431, 38.4 and 66.9 pg/mL, respectively) (*P* < 0.05). However, the concentration of other cytokines such as IL-2 were also statistically different and higher in the serum of goats in YM9 (1,234 pg/mL) and AH35 (1,043 pg/mL) groups than in CK (636 pg/mL) group (*P* < 0.05). IL-4 concentrations did not differ between the YM9 (140 pg/mL) and CK (140 pg/mL) groups; however, both were significantly higher than those observed in the AH35 group (60.8 pg/mL) (*P* < 0.05). Notably, the CK group had significantly higher anti-inflammatory cytokine IL-10 (172 pg/mL) followed by YM9 (112 pg/mL) and lastly AH35 (82.1 pg/mL) (*P* < 0.05).

### Characteristics of gene expression in dairy goats’ mammary gland

**Fig. 3:**
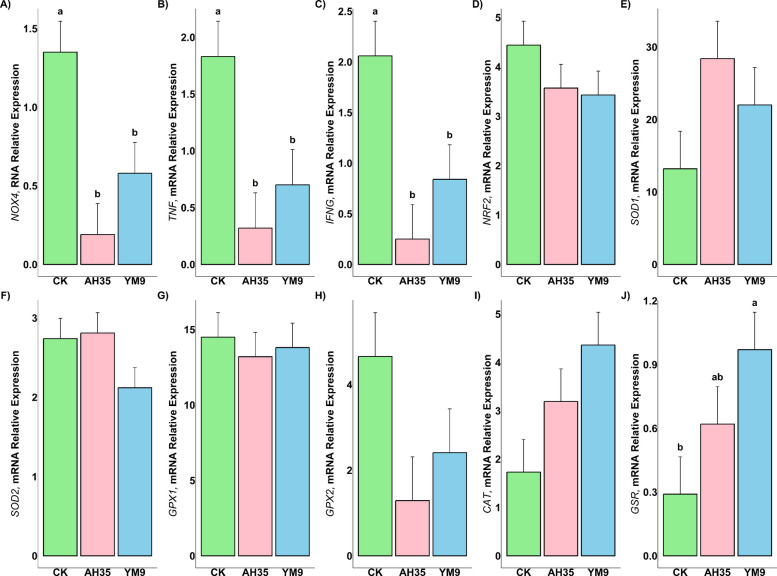
The related gene expressions of pro-inflammatory (**A**–**C**), anti-inflammatory (**D**), and antioxidant (**E**–**J**) in the mammary glands of the dairy goats *n* = 12. Different letters (a, b) above bars indicate significant differences (*P* < 0.05)

The related gene expressions of pro-inflammatory (*NOX4*,* TNF* and *IFNG*), anti-inflammatory (*NRF2*), as well as antioxidant (*SOD*1, *SOD2*,* GPX1*, *GPX2*, *CAT*, and *GSR*) in the mammary glands of the dairy goats are presented in Fig. 3. The figure revealed that dairy goats fed TMR containing silage inoculated with *Lent. buchneri* treatment (YM9 and AH35) had the relative mRNA expression of the pro-inflammatory/oxidation-related genes (*NOX4*, *TNF* and *IFNG*) been significantly downregulated (*P* < 0.05). However, YM9 group had the relative mRNA expression of the antioxidant-related *GSR* gene been significantly upregulated in the goats’ mammary gland (*P* < 0.05). The remaining *NRF2*,* SOD*1, *SOD2, GPX1*, *GPX2* and *CAT* genes were not different among the treatments (*P* > 0.05).

### Dairy goats’ rumen bacterial community

**Fig. 4:**
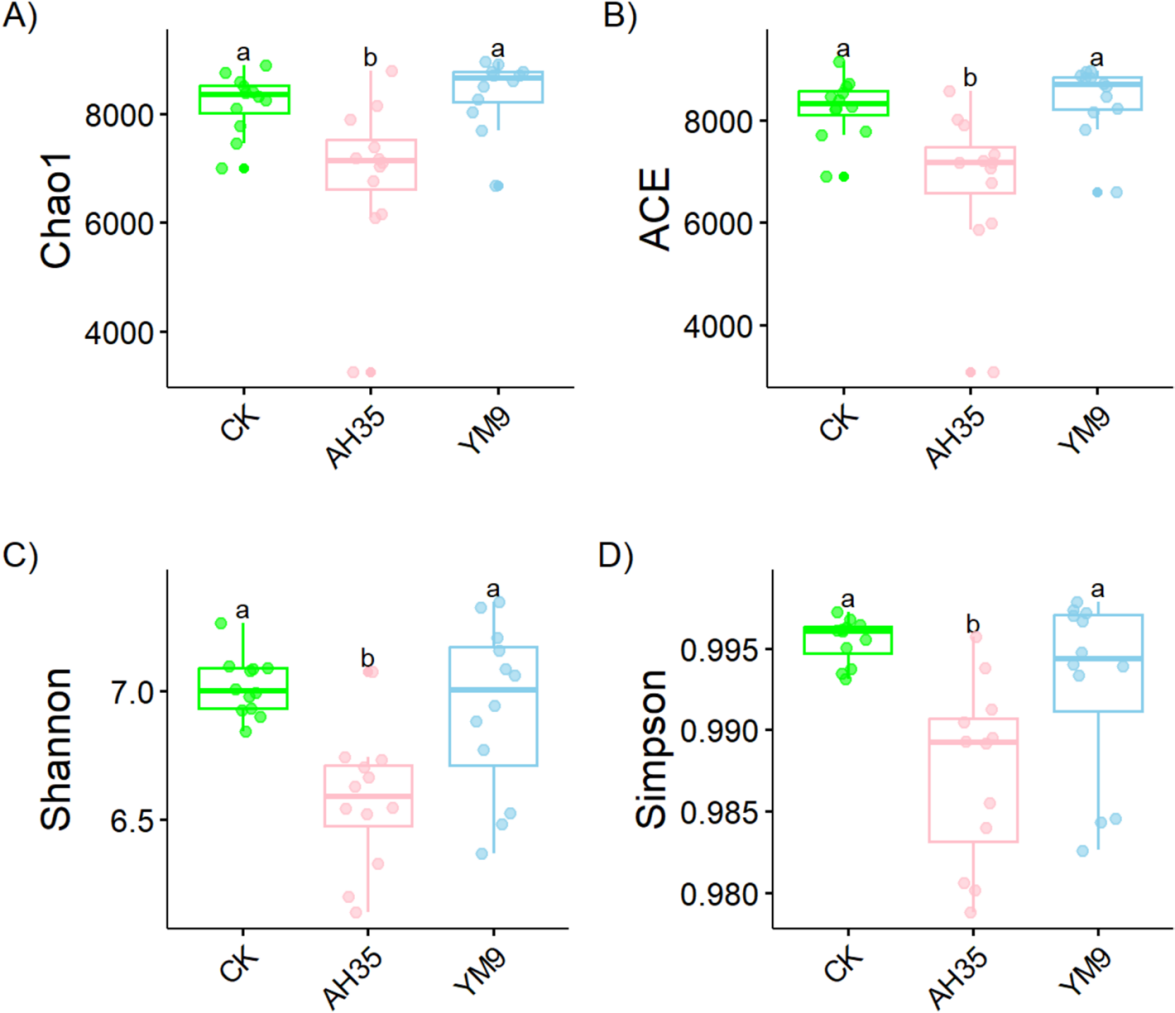
Dairy goats’ alpha diversity of rumen bacterial community (**A**–**D**) *n* = 12. Different letters (a, b) above boxes indicate significant differences (*P* < 0.05)

The alpha diversity indices including Chao1, ACE, Shannon and Simpson of rumen bacterial community of the dairy goats are presented in Fig. 4. Although the figure revealed significant differences among the different treatments in all the alpha diversity indices (*P* < 0.05), but the trend of the differences remains similar in all the indices. The values of Chao1, ACE, Shannon and Simpson were all higher in the dairy goats fed diets containing CK and YM9 silages, while those fed diets containing AH35 inoculated silage had significantly lower values for all the indices (*P* < 0.05).

**Fig. 5:**
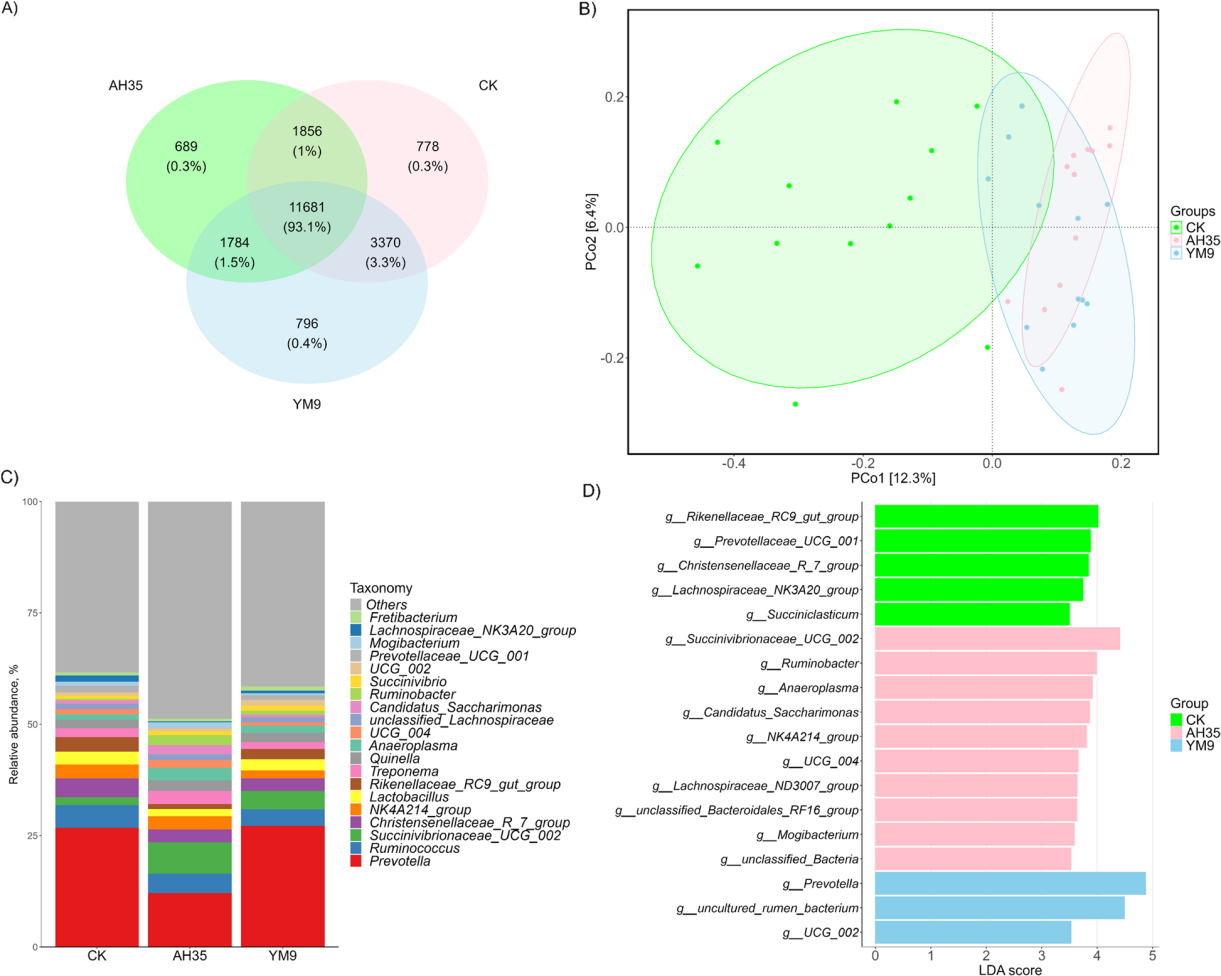
Dairy goats’ beta diversity (**A**–**D**) of rumen bacterial community

The rumen of the dairy goats in all the diet treatments shared about 93% of the bacterial OTU (Fig. 5a). However, while the CK and AH35 groups had only 0.3% unique bacterial OTU, the YM9 group had up to 0.4% unique bacterial OTU. Notably, CK and YM9 groups share 3.3% bacterial OTU which is more than 50% of the OTU each of them shared with AH35 group. Both PCoA1 and PCoA2 revealed that only 18.7% of the variation in the rumen bacterial community could be accounted (Fig. 5b). Whereas inoculated treatment groups (AH35 and YM9) were clustered on the right side, the CK group appeared to be spread largely around the left side.

The relative abundance of the dairy goats’ rumen bacterial community at genus level revealed that *Prevotella* dominated the rumen microbial community with its relative abundance in CK and YM9 groups being virtually twice than in AH35 group (Fig. 5c). There was an increased relative abundance of *Succinicvibrionaceae*_UCG_002 and decreased abundance of *Christensenellaceae*_R_7_group in AH35 and YM9 group. LEfSe analysis revealed that several of the bacterial genera had significant abundance in different dietary treatments (Fig. 5d). The genus *Prevotella* which dominated the rumen, had significant abundance only in the YM9 group. Other genera with significant abundance in the YM9 group were *Uncultured_rumen_bacterium* and *UCG_002*. Notably, the abundance of the genus *Succinicvibrionaceae* which increased in the rumen of dairy goats fed diets containing inoculated silages was found to be significant in the AH35 group.

### Spearman correlation and Mantel’s test of various parameters including top 20 bacterial genera with the mammary gland genes

**Fig. 6:**
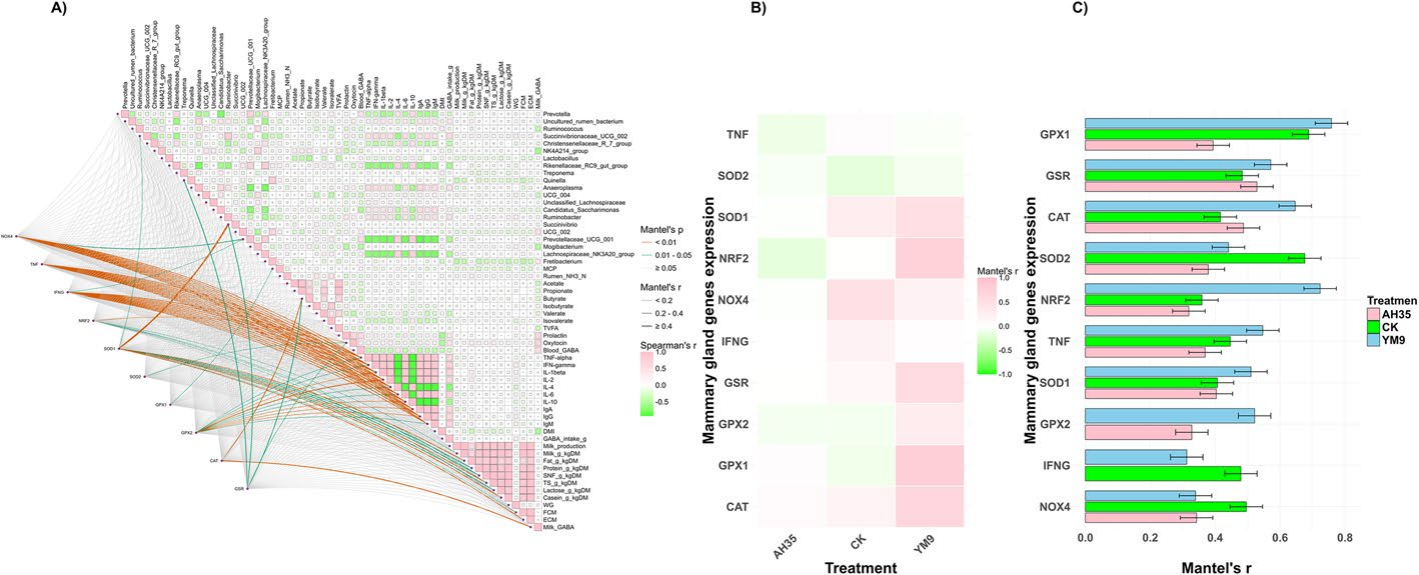
Spearman correlation and Mantel’s test of dairy goats’ rumen bacterial community and fermentation, physio-hormonal parameters and immunity status as well as performance and the mammary gland gene expression. **A** Spearman correlation and Mantel’s test. **B** Direction and strength of Mantel’s correlation among treatments. **C** Significant Mantel’s correlation among treatments

The relationships among rumen bacterial genera (top 20), fermentation parameters, GABA intake, blood GABA and lactation-related hormones, immunoglobulins and cytokines, lactation performance, milk components, and milk GABA concentration are shown in Fig. 6A and Additional file 2. There were 361 significant (*P* < 0.05) pairwise Spearman correlations among the various parameters and the top 20 genera of the bacterial community, out of which 204 were positive and 157 were negative. The genus *Prevotella* exhibits a strong negative correlation with pro-inflammatory cytokines (TNF-α, IFN-γ, IL-1β, IL-2, IL-6) and immunoglobulins (IgA, IgG, IgM). Conversely, *Prevotella* was positively correlated with the anti-inflammatory cytokine IL-10, the rumen bacterium *Rikenellaceae_RC9_gut_group*, and isovalerate. These suggest that *Prevotella* may be part of microbial community associated with reduced systemic inflammation as well as favorable metabolic conditions. On the other hand, a cluster of taxa, including *Anaeroplasma*, *Candidatus_saccharimonas*, and *Succinivibrionaceae*_*UCG_002*, shows a consistent positive correlation with pro-inflammatory cytokines (TNF-α, IFN-γ, IL-1β, IL-2, IL-6) and negative correlation with IL-10. Notably, *Uncultured_rumen_bacterium* was also positively associated with these inflammatory cytokines. This consortium was inversely associated with the *Prevotella* network. The pro-inflammatory cytokines (TNF-α, IFN-γ, IL-1β, IL-2, IL-6) exhibited an extremely high significant positive intercorrelations (*r* > 0.96, *P* < 0.001) among themselves, indicating a coordinated immune response. However, their correlation with the anti-inflammatory cytokine IL-10 was uniformly negative. The concentration of GABA in the blood was positively associated with prolactin and oxytocin, and negatively correlated with the *Mogibacterium* and *Candidatus_Saccharimonas*. The neuroendocrine hormones oxytocin and prolactin were positively correlated to each other (*r* = 0.702, *P* < 0.001), and both had positive correlation with milk GABA concentration. However, milk components such as fat, protein, lactose, casein, SNF and TS were highly intercorrelated (*r* > 0.75, *P* < 0.001) and positively associated with the milk yield.

Mantel’s test was employed to examine the relationship between mammary gland gene expression and all the parameters determined from the dairy goats including the top 20 genera of the rumen bacterial community. Overall, the strength and direction of these correlations were highly dependent on treatment (Fig. 6B). In the CK group, the parameters showed a strong positive Mantel’s correlation with *NOX4*, alongside weak positive correlations with the antioxidant genes *CAT*, *GSR*, *IFNG*, and *SOD1*. Conversely, the parameters in the CK group exhibited negative correlations with *GPX1*, *GPX2*, and *SOD2*, and no correlation was detected with *NRF2*. In AH35 group, weak positive correlations were generally exhibited with *CAT*, *GPX1*, and *NOX4*, as well as weak negative correlations with *SOD1* and *SOD2*, non-significant correlation with *IFNG*. In contrast, the YM9 treatment group demonstrated a very strong positive Mantel’s correlations with nearly all mammary gland genes with only *TNF* and *SOD2* exhibiting a weak negative correlation (Fig. 6B).

Furthermore, among the 1,710 Mantel’s correlations calculated, 95 were statistically significant (*P* < 0.05) (Additional file 2). All the significant Mantel’s correlations were positive and were pooled and visualized in Fig. 6C. In the CK group, specific rumen taxa had significant correlations (*P* < 0.05) with mammary gland’s inflammatory and antioxidant gene expression. The genus *Succinivibrionaceae_UCG_002* (*r* = 0.447, *P* = 0.033) and *Rikenellaceae_RC9_gut_group* (*r* = 0.437, *P* = 0.005) were both significantly correlated with *TNF* expression. In addition, *Treponema* was strongly associated with *IFNG* (*r* = 0.479, *P* = 0.007) and multiple antioxidant genes (*GPX1*: *r* = 0.688, *P* = 0.01; *CAT*: *r* = 0.413, *P* = 0.02), while IgA was correlated with *GPX1* (*r* = 0.475, *P* = 0.007) and *CAT* (*r* = 0.357, *P* = 0.014). In the AH35 group, *Candidatus_Saccharimonas* (*r* = 0.369, *P* = 0.006) and *Anaeroplasma* (*r* = 0.320, *P* = 0.022) were significantly correlated with mammary *TNF* expression, while *Anaeroplasma* was associated with *NOX4* (*r* = 0.332, *P* = 0.034). Acetate was correlated with both *TNF* (*r* = 0.297, *P* = 0.044) and *GPX2* (*r* = 0.293, *P* = 0.033). Blood *IFN-γ* showed strong correlations with *CAT* (*r* = 0.487, *P* = 0.004) and *GSR* (*r* = 0.424, *P* = 0.004). However, The YM9 group was characterized by significant correlations favoring homeostasis and production. The anti-inflammatory IL-10 was correlated with *SOD1* expression (*r* = 0.509, *P* = 0.003). Interestingly, nearly all milk production and composition parameters including yield, fat, protein, SNF, and lactose showed strong, significant positive correlations with the antioxidant regulator *NRF2* and the enzymes *SOD1* and *GPX1* (e.g., milk yield with *SOD1*: *r* = 0.510, *P* = 0.005). Furthermore, milk GABA content was strongly associated with this antioxidant network, correlating with *NRF2* (*r* = 0.724, *P* = 0.039), *GPX1* (*r* = 0.758, *P* = 0.026), *CAT* (*r* = 0.647, *P* = 0.006), and *GSR* (*r* = 0.571, *P* = 0.012). Consistent with the overall Mantel’s test trends, *GPX2* had no significant correlations with any parameter in the CK group, and *IFNG* had none in the AH35 group; therefore, they were not visualized in Fig. 6C.

## Discussion

### Dairy goats’ performance and milk components

Advances in developing novel and functional silage inoculants transcend improving silage fermentation efficiency [25]. Currently, LAB inoculants exert various biofunctions including enrichment of silages with bioactive compounds such as GABA [13] that are beneficial to both silage utilization and animal health. In this study, dairy goats fed silage inoculated with high GABA-producing YM9 strain (GABA enriched silage) produced a daily milk yield comparable to the CK group and significantly higher than the AH35 group despite having significantly lower DMI than the other two groups. These findings suggest that YM9-inoculated silage support improved feed utilization efficiency, which can be associated with higher dietary GABA intake in these goats. As a non-proteinogenic amino acid, GABA is linked to nutrient assimilation, modulation of metabolic stress, and endocrine activity related to lactation [1, 11, 12, 26]. Notably, the elevated milk GABA concentration in YM9 goats suggests a systemic absorption and secretion of GABA into milk, potentially reflecting both enhanced rumen fermentation efficiency and potential endocrine involvement [1, 11]. In addition to superior milk output, the higher milk components such as lactose and TS per unit DMI further indicate a probable improvement of nutrient partitioning toward milk synthesis. This could possibly be due to the marginally enhanced microbial protein synthesis as seen in the MCP of YM9 group, and its post-absorptive metabolism. Dairy goats in the YM9 group showed a decreasing tendency of NH_3_-N that conform with a marginally higher MCP which suggest a better synchronization between energy and N availability thereby resulting in efficient utilization [27]. Although overall rumen fermentation was largely unchanged, as indicated by similar pH and major volatile fatty acids across treatments, the tendency toward lower butyrate and higher branched-chain volatile fatty acids in the YM9 group may reflect subtle shifts in microbial metabolism that favor improved nutrient utilization and lactation performance [28]. The intermediate DMI and lowest milk yield, but higher milk fat, protein, and casein yield per unit of DMI in the AH35 group suggest that silage inoculation itself, rather than GABA content or DMI, conferred metabolic benefits. These benefits may arise from inoculation-driven improvements in fermentation quality, nutrient preservation, and rumen microbial efficiency, thereby enhancing post-absorptive nutrient utilization and milk component synthesis. Therefore, these findings suggest that YM9-inoculated silage enhances lactational efficiency not by increasing intake, but by optimizing nutrient utilization through improved rumen function and possibly endocrine modulation via GABA. This aligns with previous reports linking dietary GABA to improved lactational performance under stress or suboptimal intake conditions [1, 11]. However, the present interpretations remain associative rather than mechanistic demonstration.

### Dairy goats’ rumen fermentation profile

The uniformity in rumen pH and concentrations of TVFA, acetate, propionate, and butyrate across all treatments indicates that core fermentation activity of the rumen microbiota remained stable, and that neither the YM9 nor AH35 silages disrupted carbohydrate fermentation. The average rumen pH observed in all the treatments falls within the normal physiological range of 6.5–7.5 for goats [29], reflecting a well-buffered rumen environment conducive for optimal microbial activity without the risk of acidosis. Zhang et al. [17] reported a higher rumen pH values of up to 7.8 in dairy goats fed alfalfa silage. However, the significant variations in branched-chain VFAs (valerate and isovalerate) highlighted a shift in nitrogen metabolism. Unlike major VFAs derived from carbohydrate fermentation, branched-chain VFAs originate from microbial deamination of branched-chain amino acids (valine, leucine, and isoleucine) and serve as indicators of protein degradation and microbial growth precursors [30]. The marginally elevated isobutyrate concentration in the YM9 group suggests enhanced valine fermentation and potentially reflects a shift toward amino acid-utilizing microbial populations, possibly influenced by bioactive compounds in the silage such as GABA. Most of the common species known for proteolytic activities in the rumen belong to the genus *Prevotella* [31]. However, the reduction in valerate levels in the YM9 group indicates a selective rather than generalized increase in amino acid deamination which specifically favors valine metabolism while limiting leucine and isoleucine catabolism. This selective fermentation pattern implies more efficient nitrogen utilization process in the rumen, with microbial metabolism optimized toward fiber-degrading activity rather than excessive amino acid breakdown. Conversely, the higher concentrations of these branched-chain volatile fatty acids in the CK group may indicate greater proteolysis and nitrogen loss. In the YM9 group, the nitrogen-scavenging role of GABA may have contributed to lower free amino acid availability for deamination, thereby enhancing microbial protein synthesis and nitrogen retention [32]. However, because ruminal free amino acids and direct nitrogen-scavenging effects were not measured, this interpretation remains speculative. Since the TVFA concentration is similar across treatments, the altered branched-chain volatile fatty acids profile in the YM9 group reflects improved microbial nitrogen efficiency, reduced ammonia accumulation, and support a metabolism favoring microbial biomass synthesis rather than nitrogen waste. The observed patterns suggest improved microbial nitrogen efficiency in the YM9 group, which may be linked to the lower feed intake yet higher milk yield, but causal relationships need to be further investigated.

### Dairy goats’ serum GABA, prolactin and oxytocin

The higher serum GABA concentration in the YM9 group suggests enhanced systemic GABA availability, which could partly result from the higher dietary GABA intake, and increased endogenous GABA production. As a neuromodulator, GABA enhances parasympathetic activity, promoting nutrient assimilation and reducing metabolic stress in animals [33–35]. Lower stress levels facilitate more efficient nutrient partitioning toward productive functions such as lactation. Correspondingly, prolactin and oxytocin levels were elevated in YM9 goats which might have influenced the observed differences in milk production as prolactin stimulates milk synthesis by alveolar cells, while oxytocin triggers myoepithelial contractions for milk ejection [36, 37]. GABA has been shown to upregulate these lactation hormones through central and pituitary pathways [38, 39], supporting the observed improvement in milk yield despite reduced feed intake. Moreover, GABA’s ability to suppress cortisol secretion and modulate the hypothalamic–pituitary–adrenal axis [40] likely contributed to reduced catabolic stress, enhancing nutrient utilization efficiency. GABA may have contributed to reducing energy expenditure associated with stress responses, thereby redirecting metabolic resources toward anabolic processes such as milk synthesis. This might have partly contributed to the improved feed conversion efficiency observed in the YM9 group.

### Dairy goats’ serum immunoglobulins and inflammatory cytokines

The serum immune profiles of the dairy goats indicate that inoculated silages may have an influence on humoral and cytokine responses. Both AH35 and YM9 groups showed elevated IgA, IgG, and IgM, implying enhanced humoral immunity and improved mucosal defense. Notably, increased IgA may strengthen mucosal barriers, limit pathogen translocation, and support systemic metabolic stability under intensive production conditions [17]. Despite these shared humoral enhancements, the two inoculated groups displayed distinct cytokine patterns. Goats fed AH35 silage showed marked elevations in pro-inflammatory cytokines (TNF-α, IFN-γ, IL-1β, IL-6) alongside suppressed IL-4 and IL-10, reflecting a more inflammatory-skewed profile. In contrast, YM9-fed goats demonstrated moderate increases in pro-inflammatory cytokines relative to CK, but these levels were significantly lower than AH35, while IL-10 remained higher than in AH35 group. This pattern indicates controlled immune activation where the immune system became primed and vigilant without triggering excessive inflammation since no pathological signs were observed [41, 42]. These findings suggest a relatively balanced and physiologically appropriate immune stimulation, characterized by moderate pro-inflammatory activation alongside preserved IL-10 and IL-4 levels associated with Th2 responses [43], rather than the marked suppression of pro-inflammatory cytokines observed in the CK group.

The relative balance in the immune response observed in goats fed *Lent. buchneri* YM9-inoculated silage was consistent with the established role of GABA in maintaining immune homeostasis. GABA and its receptors are expressed across diverse immune cell types, where they modulate cytokine release, promote IL-10–producing macrophage phenotypes, and attenuate excessive inflammatory signaling via inhibition of NF-κB pathways [6]. These results align with growing evidence that GABAergic signaling and GABA-enriched feeds recalibrate immune responses toward greater balance that enhances B cell and antibody functions while restraining overactive pro-inflammatory cascades through IL-10–mediated regulation. B cell–derived GABA has been shown to induce IL-10⁺ macrophages that suppress CD8⁺ T-cell effector activity, thereby limiting systemic inflammation [7]. Similarly, dietary GABA supplementation in piglets increased serum IgA and IgM and elevated Th2 cytokines such as IL-4, correlating with enhanced mucosal immunity and reduced inflammatory burden [8]. Collectively, these findings support that YM9 feeding elicits a physiologically balanced immunomodulation thereby achieving robust humoral activation with a lower inflammatory cost. Although the findings remain exploratory but is likely associated with GABA-driven regulatory signaling networks and they need to be mechanistically verified.

### Gene expression of mammary gland in dairy goats

The expression profiles of inflammatory and antioxidant-related genes in the mammary gland provide further insight into the systemic effects of the silage inoculation treatments. Notably, there was remarkable downregulation of the mRNA expression of pro-inflammatory genes (*NOX4*, *TNF*, and *IFNG*). These genes are known mediators of oxidative stress and immune activation in mammary gland tissue [17]. For instance, *NOX4* promotes the generation of reactive oxygen species (ROS), while *TNF* and *IFNG* are key cytokines involved in the initiation and amplification of inflammatory responses [17, 44]. Hence, their reduced expression indicates an attenuation of oxidative and inflammatory burden, which may help sustain mammary gland tissue integrity and improve milk production efficiency.

In contrast, the upregulation of *GSR* in the YM9 group among the antioxidant-related genes suggest enhanced antioxidant defense mechanisms specific to this treatment. *GSR* plays a central role in maintaining intracellular redox balance through glutathione recycling [45]. The selective upregulation of this gene in the YM9 group may reflect an adaptive response to modulating oxidative stress, potentially mediated by the bioactivity of GABA in the YM9 silage. Notably, there were no remarkable differences in the expression of *NRF2* (a master regulator of antioxidant responses) among the treatments. These findings align with the broader physiological outcomes observed of higher milk yield and improved feed conversion efficiency in YM9 group which may be partly attributed to lower systemic inflammation and enhanced oxidative stress mitigation. Previous studies have demonstrated that enhanced antioxidant enzyme activities in various tissues, including the mammary gland modulated the *NRF2*/Keap1 axis and reduced pro-inflammatory cytokine expression [17]. Overall, the results suggest that YM9-inoculated silage supports mammary gland health by dampening oxidative stress and promoting antioxidant resilience as seen in the expression of their biomarkers genes, which might have contributed to the improved lactational performance observed in the group.

### Dairy goats’ rumen bacterial community

The differences in alpha diversity indices of the rumen bacterial community reflect distinct impacts of the silage inoculation treatments on the richness and evenness of the rumen bacterial community. Specifically, the higher alpha diversity in the CK and YM9 groups indicates that YM9 preserved or promoted microbial richness and community stability to remain similar with the control. However, the consistent lower diversity across all indices in AH35 group indicates that AH35-inoculated silage had a negative influence on the microbial diversity. Higher rumen microbial diversity is generally associated with enhanced ecosystem resilience and functional redundancy, supporting stable fermentation and nutrient utilization [46]. The reduced alpha diversity observed in the AH35 group partially explains its lower feed efficiency and lactation performance, as less diverse rumen microbiota are likely to limit metabolic versatility. Despite these diversity shifts, approximately 93% of operational taxonomic units (OTUs) were shared across all treatment groups, indicating a core rumen microbiome common to all goats. Henderson et al. [47] demonstrated in a large-scale study that, despite variation due to diet and host species, a core rumen microbiome is present across diverse ruminants. However, goats in the YM9 group had 0.1% higher proportion of unique OTUs than other treatments, suggesting that the *L. buchneri* YM9 inoculant selectively enriched minor bacterial populations not present in other groups. Additionally, sharing 3.3% of OTUs between CK and YM9 which is more than twice what each shared with AH35, further confirmed a closer microbial similarity between the CK and YM9 diets. However, PCoA reflects differences in overall community structure and relative abundance rather than simple OTU presence, showed clustering of YM9 with AH35. This suggests that silage inoculation exerted a dominant effect on the abundance and organization of the rumen microbiome, leading to similar community structures in inoculated groups despite differences in OTU overlap. In contrast, the CK group formed a distinct cluster, highlighting the absence of inoculant-driven modulation of microbial community structure.

At the genus level, *Prevotella* dominated the rumen microbiome across all groups but was almost twice as abundant in CK and YM9 goats compared to AH35. *Prevotella* is a key genus in carbohydrate and protein fermentation and is associated with efficient VFA production and nitrogen metabolism [48, 49]. Its high abundance in YM9 partly explains the enhanced feed efficiency and higher yields of milk component. McLoughlin et al. [50] posited based on their study with lambs, that feed efficiency in sheep is likely influenced by changes in the abundance of specific bacteria, rather than major overall shifts in the rumen microbiome. In another sheep feeding trial, *Prevotella* took over *Ruminococcus* to dominate the rumen after feeding the experiment diets [51]. Hence, the lower *Prevotella* abundance in AH35 goats may contribute to their reduced performance. In addition to *Prevotella*, *Succinivibrionaceae*_UCG_002 was more abundant in both inoculated groups, but was significantly enriched only in AH35, according to LEfSe analysis. This genus is associated with succinate and propionate production [52]. However, its excessive abundance may indicate a shift toward fewer but more specialized fermenters, potentially reducing ecosystem balance. On the other hand, *Christensenellacea*e_R-7_group was depleted in both AH35 and YM9, which may reflect a trade-off in microbial niche occupation related to fiber degradation [53]. The LEfSe analysis further revealed that YM9 uniquely enriched several beneficial taxa, including *Prevotella* and Uncultured_rumen_bacterium, UCG_002, pointing to selective microbial modulation by the YM9 inoculant. These microbial shifts may be associated with the observed improvements in feed conversion, nitrogen utilization, and milk synthesis efficiency in the YM9 group.

### Associations among rumen microbiota, fermentation, physio-hormonal parameters, immunity, performance, and mammary gene expression

The Spearman correlation and Mantel’s tests collectively indicate associations consistent with an integrated physiological framework encompassing *Lent. buchneri* YM9-inoculated alfalfa silage (GABA-enriched), host–microbe interactions, and lactation efficiency. YM9 induced a *Prevotella*-dominant rumen consortium that correlated with an anti-inflammatory immunometabolic profile, consistent with the known roles of *Prevotella* in carbohydrate fermentation, propionate pathways, and branched-chain VFA metabolism including isobutyrate production [49, 54]. Although *Prevotella* strains can exert either pro- or anti-inflammatory effects depending on context [55–57], the YM9-associated ecotype showed strong negative correlations with systemic pro-inflammatory cytokines (TNF-α, IFN-γ, IL-1β, IL-2, IL-6) and positive correlations with IL-10, whereas AH35 fostered an association with pro-inflammatory consortium dominated by *Anaeroplasma* and *Candidatus Saccharimonas*. These contrasting microbial ecotypes highlights the likelihood of divergent physiological outcomes, where *Prevotella*-rich, anti-inflammatory state in YM9 goats associates with improved metabolic efficiency and nitrogen retention consistent with isovalerate-favored branched-chain VFA patterns.

Mantel’s test results indicated that these systemic states were associated with distinct mammary gland transcriptional profiles. In the CK group, systemic parameters were correlated with pro-oxidative marker *NOX4* and rumen taxa such as *Succinivibrionaceae_UCG_002* as well as mammary gland *TNF*, consistent with a baseline microbially associated inflammatory profile. In contrast, the YM9 group exhibited strong positive correlations with antioxidant-related genes (*SOD1*, *GPX1*) and *NRF2* [4], with most milk traits aligning with this antioxidant-associated network. IL-10 was also correlated with *SOD1*, consistent with a lower-inflammation and antioxidant-associated mammary gland profile [58]. The AH35 group displayed a more attenuated and heterogeneous pattern, with generally weak or mixed Mantel correlations and specific associations between rumen metabolites (e.g., acetate) and pro-inflammatory mammary genes. This pattern was consistent with its higher systemic inflammation and lower production efficiency.

The correlation networks also showed that blood GABA correlated positively with prolactin, oxytocin, and milk GABA. This is consistent with previous report that hypothalamic GABA receptor activation was associated with prolactin and growth hormone in mice [59] and modulation of oxytocin neurons during lactation [60]. The association between milk GABA and mammary gland antioxidant genes (*NRF2*, *GPX1*, *CAT*, *GSR*) aligns with a report that rumen-protected GABA enhanced antioxidant status in heat-stressed dairy cows [9]. Additionally, it has been found that antioxidant-rich *Lactiplantibacillus plantarum* inoculants boost mammary antioxidant gene expression in dairy goats [17]. The observed shift toward *Prevotella* in the YM9 group is likewise consistent with established functional and production-related associations reported in the literature. Species such as *Prevotella ruminicola* and related taxa are commonly associated with degradation of non-fibre substrates and microbial protein synthesis [61], and *and Prevotella-*dominant rumen communities have been linked to higher milk yield and feed efficiency [62].

Therefore, our results suggest that feeding YM9-inoculated, GABA-enriched silage is associated with higher antioxidant capacity, altered lactogenic hormone levels, and shifts in rumen nitrogen metabolism and microbial composition, including *Prevotella* abundance, alongside changes in mammary gland gene expression and milk production. While these observations are consistent with a coordinated physiological response, the study only provides exploratory evidence of correlated responses that may inform future mechanistic studies aimed at clarifying how GABA-enriched silage influences ruminant physiology and productivity.

## Conclusion

This study demonstrates that high GABA-producing *L. buchneri* YM9-inoculated silage influences enhanced lactational efficiency in dairy goats characterized by sustained milk yield despite reduced DMI. YM9-fed goats exhibited higher serum and milk GABA levels alongside coordinated changes in rumen microbial composition, endocrine parameters, immune markers, and mammary gene expression. Specifically, the rumen of the goats in the YM9 group exhibited higher *Prevotella* enrichment and altered branched-chain VFAs. They also had elevated serum prolactin and oxytocin concentrations, with relatively enhanced immunoglobulins (IgA, IgG and IgM) and moderate inflammatory cytokines (TNF-α, IFN-γ, IL-1β and IL-6). Mammary gland expression of inflammatory related genes (*NOX4*, *TNF*, *IFNG*) were suppressed whereas *GSR* was upregulated. Correlation network analysis further revealed significant associations among GABA levels, most abundant microbial taxa, physiological indicators, and mammary gland genes. These findings present an alternative approach for dietary GABA delivery to ruminants for enhanced productivity and health, and explored association among rumen, physiological, and mammary responses that contributed to improved nutrient-use efficiency. Therefore, this study establishes a foundation for subsequent research to elucidate the effects of these mechanisms on animal health and productivity.

## Supplementary Information

Additional file 1: Table S1. Silages fermentation characteristics. Table S2. Enzyme-linked immunosorbent assay (ELISA) kits. Table S3. Primer sequences used for quantitative RT-PCR amplifications.

Additional file 2. Pearson correlation and Mantel’s test.

## Data Availability

The datasets used and/or analysed during this study are available through the corresponding author upon reasonable request.

## Abbreviations

ADF: Acid detergent fiber
ANOVA: Analysis of variance
CAT: Catalase
CP: Crude protein
CFU: Colony-forming units
DIM: Days in milk
DM: Dry matter
DMI: Dry matter intake
ECM: Energy-corrected milk
FW: Fresh weight
GABA: Gamma-aminobutyric acid
GAPDH: Glyceraldehyde-3-phosphate dehydrogenase
GPX: Glutathione peroxidase
GSR: Glutathione-disulfide reductase
HSD: Tukey’s honestly significant difference
IFN: Interferon
IFNG: Interferon gamma
Ig: Immunoglobulin
IL: Interleukin
LAB: Lactic acid bacteria
NDF: Neutral detergent fiber
NRF2: Nuclear factor erythroid 2 like 2
NH_3_-N: Ammonia nitrogen
NOX4: NADPH oxidase 4
OTU: Operational taxonomic units
PCR: Polymerase chain reaction
SNF: Solids-not-fat
SOD: Superoxide dismutase
TNF: Tumor necrosis factor
TMR: Total mixed ration
TS: Total solid
VFA: Volatile fatty acid
TVFA: Total volatile fatty acid

## References

[1] Su Y, Cheng Z, Liu W, Wu T, Wang W, Lin M. Effects of rumen-protective γ-aminobutyric acid additive on lactation performance and serum biochemistry in heat-stressed cows. Front Vet Sci. 2023;10:1228155. 10.3389/fvets.2023.1228155. 37808113 10.3389/fvets.2023.1228155 PMC10556515

[2] Bae J, Moniruzzaman M, Je H-W, Lee S, Choi W, Min T, et al. Evaluation of gamma-aminobutyric acid (GABA) as a functional feed ingredient on growth performance, immune enhancement, and disease resistance in olive flounder (*Paralichthys olivaceus*) under high stocking density. Antioxidants. 2024;13:647. 10.3390/antiox13060647. 38929086 10.3390/antiox13060647 PMC11201082

[3] Jin Z, Mendu SK, Birnir B. GABA is an effective immunomodulatory molecule. Amino Acids. 2013;45:87–94. 10.1007/s00726-011-1193-7. 22160261 10.1007/s00726-011-1193-7 PMC3680704

[4] Bhat R, Axtell R, Mitra A, Miranda M, Lock C, Tsien RW, et al. Inhibitory role for GABA in autoimmune inflammation. Proc Natl Acad Sci USA. 2010;107:2580–5. 10.1073/pnas.0915139107. 20133656 10.1073/pnas.0915139107 PMC2823917

[5] Heli Z, Hongyu C, Dapeng B, Shin TY, Yejun Z, Xi Z, et al. Recent advances of γ-aminobutyric acid: physiological and immunity function, enrichment, and metabolic pathway. Front Nutr. 2022;9:1076223. 10.3389/fnut.2022.1076223. 36618705 10.3389/fnut.2022.1076223 PMC9813243

[6] Bhandage AK, Jin Z, Korol SV, Shen Q, Pei Y, Deng Q, et al. Gaba regulates release of inflammatory cytokines from peripheral blood mononuclear cells and CD4^+^ T cells and is immunosuppressive in type 1 diabetes. EBioMedicine. 2018;30:283–94. 10.1016/j.ebiom.2018.03.019. 29627388 10.1016/j.ebiom.2018.03.019 PMC5952354

[7] Zhang B, Vogelzang A, Miyajima M, Sugiura Y, Wu Y, Chamoto K, et al. B cell-derived GABA elicits IL-10^+^ macrophages to limit anti-tumour immunity. Nature. 2021;599:471–6. 10.1038/s41586-021-04082-1. 34732892 10.1038/s41586-021-04082-1 PMC8599023

[8] Zhao Y, Wang J, Wang H, Huang Y, Qi M, Liao S, et al. Effects of GABA supplementation on intestinal SIgA secretion and gut microbiota in the healthy and ETEC-infected weanling piglets. Mediators Inflamm. 2020;2020:1–17. 10.1155/2020/7368483. 10.1155/2020/7368483 PMC7271228 32565729

[9] Cheng J, Zheng N, Sun X, Li S, Wang J, Zhang Y. Feeding rumen-protected gamma-aminobutyric acid enhances the immune response and antioxidant status of heat-stressed lactating dairy cows. J Therm Biol. 2016;60:103–8. 10.1016/j.jtherbio.2016.06.011. 27503722 10.1016/j.jtherbio.2016.06.011

[10] Zhong G, Shao D, Wang Q, Tong H, Shi S. Effects of dietary supplemented of γ-amino butyric acid on growth performance, blood biochemical indices and intestinal morphology of yellow-feathered broilers exposed to a high temperature environment. Ital J Anim Sci. 2020;19:431–8. 10.1080/1828051X.2020.1747953.

[11] Wang DM, Wang C, Liu HY, Liu JX, Ferguson JD. Effects of rumen-protected γ-aminobutyric acid on feed intake, lactation performance, and antioxidative status in early lactating dairy cows. J Dairy Sci. 2013;96:3222–7. 10.3168/jds.2012-6285. 23498023 10.3168/jds.2012-6285

[12] Wang DM, Liu HY, Wang C, Liu JX, Ferguson JD. Effects of rumen-protected gamma-aminobutyric acid on feed intake, performance and antioxidative status in transition cows. Livest Sci. 2013;153:66–72. 10.1016/j.livsci.2013.01.012. 10.3168/jds.2012-6285 23498023

[13] Usman S, Zhang J, Zhu J, Zhang Y, Xu D, Dele PA, et al. Enrichment of corn and alfalfa silage with γ-aminobutyric acid through inoculation with a screened high producing *Lentilactobacillus buchneri* strain. Anim Feed Sci Tech. 2024;314:116016. 10.1016/j.anifeedsci.2024.116016.

[14] Wang YL, Zhang ZH, Wang WK, Wu QC, Zhang F, Li WJ, et al. The effect of γ-aminobutyric acid addition on in vitro ruminal fermentation characteristics and methane production of diets differing in forage-to-concentrate ratio. Fermentation. 2023;9:105. 10.3390/fermentation9020105.

[15] Novac CS, Nadăs GC, Matei IA, Bouari CM, Kalmár Z, Crăciun S, et al. Milk pathogens in correlation with inflammatory, oxidative and nitrosative stress markers in goat subclinical mastitis. Animals. 2022;12:3245. 10.3390/ani12233245. 10.3390/ani12233245 PMC9740090 36496766

[16] Khan MZ, Huang B, Kou X, Chen Y, Liang H, Ullah Q, et al. Enhancing bovine immune, antioxidant and anti-inflammatory responses with vitamins, rumen-protected amino acids, and trace minerals to prevent periparturient mastitis. Front Immunol. 2024. 10.3389/fimmu.2023.1290044. 10.3389/fimmu.2023.1290044 PMC10800369 38259482

[17] Zhang Y, Usman S, Li Q, Li F, Zhang X, Nussio LG, et al. Effects of antioxidant-rich Lactiplantibacillus plantarum inoculated alfalfa silage on rumen fermentation, antioxidant and immunity status, and mammary gland gene expression in dairy goats. J Anim Sci Biotechnol. 2024. 10.1186/s40104-023-00977-3. 10.1186/s40104-023-00977-3 PMC10802014 38247012

[18] National Research Council. Nutrient requirements of small ruminants: sheep, goats, cervids, and new world camelids. Washington, D.C.: National Academies Press; 2007. p. 11654. 10.17226/11654.

[19] Usman S, Li F, An D, Shou N, Deng J, Zhang Y, et al. Lignocellulose degradation and enzymatic hydrolysis of soybean incorporated sorghum silage inoculated with feruloyl-esterase producing *Lactobacillus plantarum*. Fermentation. 2022;8:70. 10.3390/fermentation8020070.

[20] AOAC. Official methods of the official analytical chemists. 17th ed. Horwitz W, editor. Washington DC: Association of Official Analytical Chemists; 2002.

[21] van Soest PJ, Robertson JB, Lewis BA. Methods for dietary fiber, neutral detergent fiber, and nonstarch polysaccharides in relation to animal nutrition. J Dairy Sci. 1991;74:3583–97. 10.3168/jds.S0022-0302(91)78551-2. 1660498 10.3168/jds.S0022-0302(91)78551-2

[22] Li F, Usman S, Huang W, Jia M, Kharazian ZA, Ran T, et al. Effects of inoculating feruloyl esterase-producing *Lactiplantibacillus plantarum* A1 on ensiling characteristics, in vitro ruminal fermentation and microbiota of alfalfa silage. J Anim Sci Biotechnol. 2023;14:43. 10.1186/s40104-023-00837-0. 36915166 10.1186/s40104-023-00837-0 PMC10012570

[23] Broderick GA, Kang JH. Automated simultaneous determination of ammonia and total amino acids in ruminal fluid and in vitro media. J Dairy Sci. 1980;63:64–75. 10.3168/jds.S0022-0302(80)82888-8. 7372898 10.3168/jds.S0022-0302(80)82888-8

[24] Liu C, Cui Y, Li X, Yao M. Microeco: an R package for data mining in microbial community ecology. FEMS Microbiol Ecol. 2021;97:fiaa255. 10.1093/femsec/fiaa255. 33332530 10.1093/femsec/fiaa255

[25] Guo X, Xu D, Li F, Bai J, Su R. Current approaches on the roles of lactic acid bacteria in crop silage. Microb Biotechnol. 2023;16:67–87. 10.1111/1751-7915.14184. 36468295 10.1111/1751-7915.14184 PMC9803335

[26] Zhang YT, Yang Y, Bu DP, Ma L. The effects of gamma-aminobutyric acid on growth performance, diarrhoea, ruminal fermentation, and antioxidant capacity in pre-weaned calves. Animal. 2025;19:101493. 10.1016/j.animal.2025.101493. 40279853 10.1016/j.animal.2025.101493

[27] Yang JY, Seo J, Kim HJ, Seo S, Ha JK. Nutrient synchrony: is it a suitable strategy to improve nitrogen utilization and animal performance? Asian Australas J Anim Sci. 2010;23:972–9. 10.5713/ajas.2010.r.04.

[28] Mitchell KE, Socha MT, Kleinschmit DH, Moraes LE, Roman-Garcia Y, Firkins JL. Assessing milk response to different combinations of branched-chain volatile fatty acids and valerate in Jersey cows. J Dairy Sci. 2023;106:4018–29. 10.3168/jds.2022-22545. 37059661 10.3168/jds.2022-22545

[29] Bayne JE, Edmondson MA. Diseases of the gastrointestinal system. Sheep, goat, and cervid medicine. Elsevier; 2021:63–96. 10.1016/B978-0-323-62463-3.00014-1.

[30] Dehority BA, Johnson RR, Bentley OG, Moxon AL. Studies on the metabolism of valine, proline, leucine and isoleucine by rumen microorganisms *in vitro*. Arch Biochem Biophys. 1958;78:15–27. 10.1016/0003-9861(58)90310-2. 13595899 10.1016/0003-9861(58)90310-2

[31] Patra AK, Yu Z. Genomic insights into the distribution of peptidases and proteolytic capacity among *Prevotella* and *Paraprevotella* species. Microbiol Spectr. 2022;10:e02185-21. 10.1128/spectrum.02185-21. 10.1128/spectrum.02185-21 PMC9045265 35377228

[32] Sulieman S. Does GABA increase the efficiency of symbiotic N_2_ fixation in legumes? Plant Signal Behav. 2011;6:32–6. 10.4161/psb.6.1.14318. 21307661 10.4161/psb.6.1.14318 PMC3122002

[33] Auteri M, Zizzo M, Serio R. The GABAergic system and the gastrointestinal physiopathology. Curr Pharm Des. 2015;21:4996–5016. 10.2174/1381612821666150914121518. 26365138 10.2174/1381612821666150914121518

[34] Tropskaya NS, Gurman YuV, Popova TS, Kanibolotsky AA. Gastroprotective effect of GABA in metabolic stress. Bull Exp Biol Med. 2024;177:301–6. 10.1007/s10517-024-06178-w. 39126542 10.1007/s10517-024-06178-w

[35] Fujibayashi M, Kamiya T, Takagaki K, Moritani T. Activation of autonomic nervous system activity by the oral ingestion of GABA. J Jpn Soc Nutr Food Sci. 2008;61:129–133. 10.4327/jsnfs.61.129.

[36] Bruckmaier RM, Blum JW. Oxytocin release and milk removal in ruminants. J Dairy Sci. 1998;81:939–49. 10.3168/jds.S0022-0302(98)75654-1. 9594382 10.3168/jds.S0022-0302(98)75654-1

[37] Neville MC, McFadden TB, Forsyth I. Hormonal regulation of mammary differentiation and milk secretion. J Mammary Gland Biol Neoplasia. 2002;7:49–66. 10.1023/A:1015770423167. 12160086 10.1023/a:1015770423167

[38] Nakayama Y, Hattori N, Otani H, Inagaki C. γ-Aminobutyric acid (GABA)-C receptor stimulation increases prolactin (PRL) secretion in cultured rat anterior pituitary cells. Biochem Pharmacol. 2006;71:1705–10. 10.1016/j.bcp.2006.03.014. 10.1016/j.bcp.2006.03.014 16677614

[39] Fjalland B, Christensen JD, Grell S. GABA receptor stimulation increases the release of vasopressin and oxytocin *in vitro*. Eur J Pharmacol. 1987;142:155–8. 10.1016/0014-2999(87)90667-4. 2826174 10.1016/0014-2999(87)90667-4

[40] Camille Melón L, Maguire J. GABAergic regulation of the HPA and HPG axes and the impact of stress on reproductive function. J Steroid Biochem. 2016;160:196–203. 10.1016/j.jsbmb.2015.11.019. 10.1016/j.jsbmb.2015.11.019 PMC4861672 26690789

[41] Al-Qahtani AA, Alhamlan FS, Al-Qahtani AA. Pro-inflammatory and anti-inflammatory interleukins in infectious diseases: a comprehensive review. Trop Med Infect Dis. 2024;9:13. 10.3390/tropicalmed9010013. 38251210 10.3390/tropicalmed9010013 PMC10818686

[42] Arango Duque G, Descoteaux A. Macrophage cytokines: involvement in immunity and infectious diseases. Front Immunol. 2014. 10.3389/fimmu.2014.00491. 10.3389/fimmu.2014.00491 PMC4188125 25339958

[43] Berger A. Science commentary: Th1 and Th2 responses: what are they? BMJ. 2000;321:424–424. 10.1136/bmj.321.7258.424. 10938051 10.1136/bmj.321.7258.424 PMC27457

[44] Basuroy S, Tcheranova D, Bhattacharya S, Leffler CW, Parfenova H. Nox4 NADPH oxidase-derived reactive oxygen species, via endogenous carbon monoxide, promote survival of brain endothelial cells during TNF-α-induced apoptosis. Am J Physiol-Cell Ph. 2011;300:C256–65. 10.1152/ajpcell.00272.2010. 10.1152/ajpcell.00272.2010 PMC3043629 21123734

[45] Couto N, Wood J, Barber J. The role of glutathione reductase and related enzymes on cellular redox homoeostasis network. Free Radic Biol Med. 2016;95:27–42. 10.1016/j.freeradbiomed.2016.02.028. 26923386 10.1016/j.freeradbiomed.2016.02.028

[46] Weimer PJ. Redundancy, resilience, and host specificity of the ruminal microbiota: implications for engineering improved ruminal fermentations. Front Microbiol. 2015. 10.3389/fmicb.2015.00296. 10.3389/fmicb.2015.00296 PMC4392294 25914693

[47] Henderson G, Cox F, Ganesh S, Jonker A, Young W, Attwood GT, et al. Rumen microbial community composition varies with diet and host, but a core microbiome is found across a wide geographical range. Sci Rep. 2015;5:14567. 10.1038/srep14567. 26449758 10.1038/srep14567 PMC4598811

[48] Bi Y, Zeng S, Zhang R, Diao Q, Tu Y. Effects of dietary energy levels on rumen bacterial community composition in Holstein heifers under the same forage to concentrate ratio condition. BMC Microbiol. 2018;18:69. 10.1186/s12866-018-1213-9. 29996759 10.1186/s12866-018-1213-9 PMC6042446

[49] Betancur-Murillo CL, Aguilar-Marín SB, Jovel J. *Prevotella*: a key player in ruminal metabolism. Microorganisms. 2022;11:1. 10.3390/microorganisms11010001. 36677293 10.3390/microorganisms11010001 PMC9866204

[50] McLoughlin S, Spillane C, Claffey N, Smith PE, O’Rourke T, Diskin MG, et al. Rumen microbiome composition is altered in sheep divergent in feed efficiency. Front Microbiol. 2020;11:1981. 10.3389/fmicb.2020.01981. 32983009 10.3389/fmicb.2020.01981 PMC7477290

[51] Usman S, Zhang Y, Abdelraheem N, Wu P, Umar SJ, Aderinboye RY, et al. Improving sorghum silage utilization by soybean incorporation and *Lactiplantibacillus plantarum* A1 inoculation: effect on in vitro digestibility and sheep performance. Anim Feed Sci Tech. 2025;116613. 10.1016/j.anifeedsci.2025.116613.

[52] Daghio M, Ciucci F, Buccioni A, Cappucci A, Casarosa L, Serra A, et al. Correlation of breed, growth performance, and rumen microbiota in two rustic cattle breeds reared under different conditions. Front Microbiol. 2021;12:652031. 10.3389/fmicb.2021.652031. 33995309 10.3389/fmicb.2021.652031 PMC8117017

[53] Fan Q, Wanapat M, Hou F. Rumen bacteria influence milk protein yield of yak grazing on the Qinghai-Tibet plateau. Anim Biosci. 2021;34:1466–78. 10.5713/ab.20.0601. 33332947 10.5713/ab.20.0601 PMC8495338

[54] Xin H, Khan NA, Liu X, Jiang X, Sun F, Zhang S, et al. Profiles of odd- and branched-chain fatty acids and their correlations with rumen fermentation parameters, microbial protein synthesis, and bacterial populations based on pure carbohydrate incubation in vitro. Front Nutr. 2021;8:733352. 10.3389/fnut.2021.733352. 34631768 10.3389/fnut.2021.733352 PMC8492898

[55] Bertelsen A, Elborn JS, Schock BC. Microbial interaction: *Prevotella* spp. reduce P. aeruginosa induced inflammation in cystic fibrosis bronchial epithelial cells. J Cyst Fibros. 2021;20:682–91. 10.1016/j.jcf.2021.04.012. 34112603 10.1016/j.jcf.2021.04.012

[56] Rodrigues GSP, Cayres LCF, Gonçalves FP, Takaoka NNC, Lengert AH, Tansini A, et al. Detection of increased relative expression units of Bacteroides and Prevotella, and decreased *Clostridium Leptum* in stool samples from Brazilian rheumatoid arthritis patients: a pilot study. Microorganisms. 2019;7:413. 10.3390/microorganisms7100413. 31581593 10.3390/microorganisms7100413 PMC6843655

[57] Iljazovic A, Roy U, Gálvez EJC, Lesker TR, Zhao B, Gronow A, et al. Perturbation of the gut microbiome by *Prevotella* spp. enhances host susceptibility to mucosal inflammation. Mucosal Immunol. 2021;14:113–24. 10.1038/s41385-020-0296-4. 32433514 10.1038/s41385-020-0296-4 PMC7790746

[58] Sordillo LM. Nutritional strategies to optimize dairy cattle immunity. J Dairy Sci. 2016;99:4967–82. 10.3168/jds.2015-10354. 10.3168/jds.2015-10354 26830740

[59] Willoughby JO, Jervois PM, Menadue MF, Blessing WW. Activation of GABA receptors in the hypothalamus stimulates secretion of growth hormone and prolactin. Brain Res. 1986;374:119–25. 10.1016/0006-8993(86)90400-2. 3013363 10.1016/0006-8993(86)90400-2

[60] Lee SW, Kim YB, Kim JS, Kim WB, Kim YS, Han HC, et al. GABAergic inhibition is weakened or converted into excitation in the oxytocin and vasopressin neurons of the lactating rat. Mol Brain. 2015;8:34. 10.1186/s13041-015-0123-0. 26017151 10.1186/s13041-015-0123-0 PMC4446001

[61] Kim JN, Méndez–García C, Geier RR, Iakiviak M, Chang J, Cann I, et al. Metabolic networks for nitrogen utilization in *Prevotella ruminicola* 23. Sci Rep. 2017;7:7851. 10.1038/s41598-017-08463-3. 28798330 10.1038/s41598-017-08463-3 PMC5552732

[62] Yang J, Li Y, Sun M, Guo S, Lin P, Wang A, et al. Understanding the differences in rumen bacteria and their impact on dairy cows’ production performance: a review. Anim Nutr. 2025;22:259–79. 10.1016/j.aninu.2025.04.006. 40896480 10.1016/j.aninu.2025.04.006 PMC12391802

