# Supplementary material for: Effects of GABA-enriched alfalfa silage on rumen microbiota, lactation hormones, immunity, and mammary gland gene expression, alongside lactation performance in dairy goats
Source: J Anim Sci Biotechnol. 2026 May 27;17:101. doi: 10.1186/s40104-026-01408-9 (PMC13214404; doi:10.1186/s40104-026-01408-9)
Supplement: Supplementary file 1 — Additional file 1: Table S1. Silages fermentation characteristics. Table S2. Enzyme-linked immunosorbent assay (ELISA) kits. Table S3. Primer sequences used for quantitative RT-PCR amplifications. [file 40104_2026_1408_MOESM1_ESM.docx]

**Additional file 1:**

Table S1: Silages fermentation characteristics.

Table S2: Enzyme-linked immunosorbent assay (ELISA) kits

Table S3: Primer sequences used for quantitative RT-PCR amplifications.

**Table S1** Nutrient and chemical composition of fresh alfalfa

| **Item** | **Treatments** | | | **SEM** | **P-value** |
| --- | --- | --- | --- | --- | --- |
|  | **CK** | **AH35** | **YM9** |  |  |
| pH | 3.81^a^ | 3.69^b^ | 3.78^a^ | 0.023 | 0.007 |
| LA, g/kg DM | 43.34a | 25.29^b^ | 25.67^b^ | 1.509 | 0.000 |
| AA, g/kg DM | 72.60 | 66.45 | 62.32 | 4.678 | 0.329 |
| PA, g/kg DM | 44.98 | 46.53 | 45.30 | 6.707 | 0.985 |
| BA, g/kg DM | 3.64 | 3.79 | 3.78 | 0.343 | 0.942 |

^a,b^ Means on the same row having different superscripts are significantly different (P < 0.05)

SEM = Standard error of the mean

DM = dry matter; LA = Lactic acid; AA = Acetic acid; PA = Propionic acid; BA = Butyric acid.

**Table S2:** Enzyme-linked immunosorbent assay (ELISA) kits

| **Product Catalog Number** | **Description** |
| --- | --- |
| YJ601136X | Goat Immunoglobulin A (IgA) ELISA Kit |
| YJ600787X | Goat Immunoglobulin G (IgG) ELISA Kit |
| YJ600740X | Goat Immunoglobulin M (IgM) ELISA Kit |
| YJ605300X | Goat Interleukin-1β (IL-1β) ELISA Kit |
| YJ600089X | Goat Interleukin-2 (IL-2) ELISA Kits |
| YJ602532X | Goat Interleukin-4 (IL-4) ELISA Kit |
| YJ602183X | White Blood Cell Interleukin 6 (IL-6) ELISA |
| YJ601834X | Goat Interleukin-10 (IL-10) ELISA Kit |
| YJ602881X | Goat γ Interferon (IFN-γ) ELISA Kits |
| YJ600438X | Goat Tumor Necrosis Factor α (TNF-α) ELISA Kit |
| YJ605324X | Goat Gamma-Aminobutyric Acid (GABA) ELISA Kits |
| YJ599391X | Goat Prolactin (PRL) |
| YJ599042X | Goat Oxytocin (OT) Assay Kit |

All kits are for research use only

**Table S3** Primer sequences used for quantitative RT-PCR amplifications

| **Gene** | **Primer sequences (5’-3’)** | **Accession number** | **Product size, bp** |
| --- | --- | --- | --- |
| *NFE2L2* | (F) CTGTTCTCTGCTGTCAAGGGA | NM_001314327.1 | 221 |
|  | (R) ACTCGCCGGTCTCTTCATCT |  |  |
| *SOD1* | (F) AGAGAGGCATGTTGGAGACC | NM_001285550.1 | 160 |
|  | (R) TCCACCTCTGCCCAAGTCAT |  |  |
| *SOD2* | (F) ACCCAAAGGGGAATTGCTGG | XM_018053428.1 | 227 |
|  | (R) CATGCTCCCACACGTCAATC |  |  |
| *GPX1* | (F) TCCTTGTTCTTCGAGTCCGC | XM_005695962.3 | 209 |
|  | (R) CCTCAGAGCGATGCTACGTT |  |  |
| *GPX2* | (F) CTCAAGTATGTCCGCCCTGG | XM_005685982.3 | 135 |
|  | (R) CGGGTCGTCATAAGGGTAGG |  |  |
| *GSR* | (F) ACACTGCTGTCCACTCTGAA | XM_018041988.1  XM_018041989.1 | 111 |
|  | (R) TCACATAGGCATCCCGCTTT |  |  |
| *CAT* | (F) CTGGAACATAGGACCCGCTT | XM_005690077.3 | 137 |
|  | (R) GCAATGTTCTCACACAGGCG |  |  |
| *NOX4* | (F) GGGATTGTGTCTAAGCAGAGC | XM_005699426.3  XM_018043547.1 | 136 |
|  | (R) CAATCTTCTGGTTCTCCGGCT |  |  |
| *TNF* | (F) CCCAGAGGGAAGAGCAGTC | NM_001286442.1 | 168 |
|  | (R) TGAGGGCATTGGCATACGAG |  |  |
| *IFNG* | (F) GATCCAGCGCAAAGCCATAAA | NM_001285682.1 | 109 |
|  | (R) TCTCCGGCCTCGAAAGAGATT |  |  |
| *GAPDH* | (F) GTCCGTTGTGGATCTGACCTG | XM_005680968.3 | 165 |
|  | (R) AAGGTAGAAGAGTGAGTGTCGC |  |  |

*NRF2* = nuclear factor erythroid 2 like 2; *SOD1* = superoxide dismutase 1; *SOD2* = superoxide dismutase 2; *GPX1* = glutathione peroxidase 1; *GPX2* = glutathione peroxidase 2; *GSR* = glutathione-disulfide reductase; *CAT* = catalase; *NOX4* = NADPH oxidase 4; *TNF* = tumor necrosis factor; *IFNG* = interferon gamma; *GAPDH* = glyceraldehyde-3-phosphate dehydrogenase.

F = Forward; R = Reverse.
